# Effects of the PPAR*α* Agonist and Widely Used Antihyperlipidemic Drug Gemfibrozil on Hepatic Toxicity and Lipid Metabolism

**DOI:** 10.1155/2010/681963

**Published:** 2010-10-04

**Authors:** Michael L. Cunningham, Bradley J. Collins, Milton R. Hejtmancik, Ronald A. Herbert, Gregory S. Travlos, Molly K. Vallant, Matthew D. Stout

**Affiliations:** National Toxicology Program, National Institute of Environmental Health Sciences, National Institutes of Health, 111 Alexander Drive, Research Triangle Park, NC 27709, USA

## Abstract

Gemfibrozil is a widely prescribed hypolipidemic agent in humans and a peroxisome proliferator and liver carcinogen in rats. Three-month feed studies of gemfibrozil were conducted by the National Toxicology Program (NTP) in male Harlan Sprague-Dawley rats, B6C3F1 mice, and Syrian hamsters, primarily to examine mechanisms of hepatocarcinogenicity. There was morphologic evidence of peroxisome proliferation in rats and mice. Increased hepatocyte proliferation was observed in rats, primarily at the earliest time point. Increases in peroxisomal enzyme activities were greatest in rats, intermediate in mice, and least in hamsters. These studies demonstrate that rats are most responsive while hamsters are least responsive. These events are causally related to hepatotoxicity and hepatocarcinogenicity of gemfibrozil in rodents via peroxisome proliferator activated receptor-*α* (PPAR*α*) activation; however, there is widespread evidence that activation of PPAR*α* in humans results in expression of genes involved in lipid metabolism, but not in hepatocellular proliferation.

## 1. Introduction

Gemfibrozil is a nonhalogenated derivative in the class of drugs called fibrates that include clofibrate, fenofibrate, and ciprofibrate. Since its approval by the FDA in 1982, it has been used extensively as a lipid-regulating drug and is an effective treatment of hypertriglyceridemia and hypercholesterolemia. The results of two clinical trials demonstrate that gemfibrozil has proven to be a valuable therapeutic agent in the control of coronary heart disease [1, 2]. It appears that gemfibrozil exerts hypolipidemic effects by decreasing the concentration of triglycerides [2] and low-density lipoprotein cholesterol (“bad” cholesterol) [3] and raising the concentration of high-density lipoprotein-cholesterol (“good” cholesterol) [2, 3]. 

In rodents, gemfibrozil and other fibrates are peroxisome proliferators, inducing a syndrome that includes enlarged livers associated with an increased number and size of hepatic peroxisomes and induction of peroxisomal and microsomal fatty acid-oxidizing enzymes including acyl CoA oxidase, carnitine acetyltransferase, and cytochrome P450 4A [4–7]. In addition to fibrates, peroxisome proliferators include selected herbicides, phthalate ester plasticizers, and endogenous long chain fatty acids [5, 8]. Peroxisome proliferators are associated with hepatocarcinogenicity in rodents. Studies with several peroxisome proliferators, including Wy-14,643 ([4-chloro-6-(2,3-xylidino)-2-pyrimidinylthio]acetic acid; the prototype peroxisome proliferator), di (2-ethylhexyl) phthalate and gemfibrozil, and clofibrate have demonstrated carcinogenicity rodents [9–14]. 

The basis for understanding the biology of peroxisome proliferation in rodents and humans began with the discovery of the peroxisome proliferator activated receptor-*α* (PPAR*α*) in 1990 [15]. Agonists for the PPAR*α* were found to induce a battery of genes, resulting in peroxisome proliferation in the cytoplasm of rodent liver, which increased lipid catabolism via induction of peroxisomal fatty acyl-CoA *β*-oxidation. In humans, fibrates including gemfibrozil bind PPAR*α* with high affinity, producing reduction in plasma triglycerides and increased HDL concentrations [16]. These effects are thought to result from reducing apoCIII expression and induction of apolipoprotein-AI and AII expression in humans, which are under control of PPAR*α*, and not by proliferation of peroxisomes which occurs in rodents [17]. The molecular basis of differences in response to the hepatic effects of peroxisome proliferators is hypothesized to be a combination of quantitative differences in the hepatic expression of PPAR*α* and qualitative differences in the pattern or functionality of the downstream events that are regulated by the receptor [18, 19]. 

Although the biochemical and physiologic effects associated with hepatic peroxisome proliferation are thought to play a role in the hepatic toxicity and carcinogenicity in sensitive species of rodents, the mechanism of peroxisome proliferator-induced tumorigenesis and the nature of its species-selectivity are not understood [20–22]. The results of a limited number of published studies suggest that gemfibrozil is not mutagenic [12, 23]. As a result, the observed hepatocarcinogenicity is thought to be the result of indirect mechanisms. Mechanisms of PPAR*α*-induced hepatocarcinogenicity have been recently reviewed [24]. Activation results in increase cell proliferation and decreased apoptosis. PPAR*α*-induced oxidative stress may contribute to cell proliferation via increased signaling or may damage DNA, resulting in the initiation of carcinogenesis; the data for peroxisome proliferator-induced DNA damage are conflicting [25, 26]. Peroxisome proliferator-induced oxidative stress is thought to occur in the rodent because treatment of rodents causes large increases in the activity of the hydrogen peroxide producing peroxisomal *β*-oxidation enzymes while causing only minimal increases in the activity of peroxisomal catalase and decreased activity of glutathione peroxidase [27–29]. One study with Wy-14,643 revealed that hepatocarcinogenicity appears to correlate better with cell proliferation rather than peroxisome proliferation [30]. PPAR*α* null mice have been used to evaluate the role of PPAR*α* in rodent hepatocarcinogenicity. Wy-14,643 hepatocarinogenicity was observed in wild type mice, but not in null mice [31, 32]. In contrast, following exposure to di (2-ethylhexyl) phthalate, more liver tumors were observed in PPARnull mice compared to wild type mice [33], suggesting that PPAR-independent mechanisms may also be active in the hepatocarcinogenicity of some peroxisome proliferators. Recently, Gonzalez and colleagues have published a series of studies in wild type and humanized PPAR*α* mice [25, 26, 32, 34, 35]. These studies demonstrate that the humanized PPAR*α* mice are resistant to hepatocellular proliferation [25] and tumors [32] following exposure to Wy-14,643. In contrast, genes involved in peroxisomal and mitochondrial *β*-oxidation are induced in the wild type and humanized mice. These authors have concluded that the observed differences in the hepatocellular response are the result of differences in the disposition of let-7C microRNA (miRNA) and c-myc expression. In the wild type mice, let-7C miRNA is downregulated, resulting in the increased expression of c-myc, hepatocellular proliferation, and tumors [26, 34, 35]. In contrast, neither downregulation of let-7C miRNA nor increased expression of c-myc occurs in humanized PPAR*α* mice, resulting in a lack of hepatocellular proliferation and tumors. These data may explain the difference in PPAR*α*-mediated effects between rodents and humans.

The National Toxicology Program (NTP) conducted a series of 3-month feed studies in male Harlan Sprague Dawley rats, B6C3F1 mice, and Syrian hamsters to evaluate mechanisms of hepatocarcinogenicity of peroxisome proliferators; Wy-14,643 [36], gemfibrozil, dibutyl phthalate, and 2,4-dichlorophenoxyacetic acid. Gemfibrozil was included in this initiative because it interacts with the PPAR*α* in rodents and humans as a mechanism of its pharmacological activity, and it induces hepatomegaly, peroxisome proliferation, and hepatocellular tumors in rodents. It was also of interest to evaluate whether these adverse effects were relevant to humans taking this therapeutic agent chronically. Rats and mice are commonly used in studies examining peroxisome proliferators and males are typically more sensitive than females. Hamsters were included because this species, like humans, is believed to be relatively resistant to the hepatotoxicity and carcinogenicity of peroxisome proliferators [37]. In addition to standard endpoints, the studies included assessments of hepatocyte cell proliferation, peroxisomal enzyme analysis, and analysis of lipid levels. Several investigators were awarded RO3 grants to study mechanistic aspects of peroxisome proliferator-induced hepatocarcinogenesis using tissues available from these studies [38–44]. The purpose of this manuscript is to present the effects of gemfibrozil on hepatic toxicity and lipid metabolism following exposure of rats, mice, and hamsters following subchronic exposure in feed, in the context of the NTP studies of Wy-14,643 [36].

## 2. Materials and Methods

### 2.1. Chemical and Dose Formulations

Gemfibrozil was obtained from Sigma Chemical Company (St. Louis, MO) in three lots. Lot 18F0334 was identified as gemfibrozil by infrared spectroscopy (IR) and proton nuclear magnetic resonance spectroscopy (NMR). Purity was determined to be >99% by high performance liquid chromatography (HPLC). Lot 02H0074 was found to be 98.7% pure by HPLC. Lots 18F0334 and 02H0074 were combined prior to the study and renamed as lot S040794. Purity of the combined lot was determined to be >99% by HPLC. A third lot, 104H0551, was identified by IR. Prior to the study the purity of lot S040794 and lot 104H0551 relative to a frozen reference sample of each lot was determined by HPLC to be 103.4% and 99%, respectively. Both of these lots were used in the 90-day studies. To ensure stability, the bulk chemical was stored in amber glass bottles sealed with Teflon-lined lids or sealed buckets lined with double Teflon bags, protected from light, at room temperature. During the studies, periodic reanalyses against frozen reference samples using HPLC revealed no degradation of the bulk chemical. Dose formulations were prepared by mixing gemfibrozil with feed and were stored in plastic buckets at approximately 5°C for up to 3 weeks. Homogeneity of selected dose formulations was confirmed by HPLC. Dose formulations were analyzed at the beginning, midpoint, and end of the studies. Of the dose formulations analyzed for rats, mice, and hamsters, 96% (26/27) were within 10% of the target concentrations.

### 2.2. Animals and Animal Maintenance

The studies were conducted at Battelle Columbus Laboratories (Columbus, OH) in compliance with Food and Drug Administration Good Laboratory Practice Regulations (21 CFR, Part 58). Male Sprague-Dawley rats were obtained from Harlan Sprague-Dawley, Inc. (Indianapolis, IN). Male B6C3F1 mice were obtained from Taconic Farms, Inc. (Germantown, NY). Male Syrian hamsters were obtained from Frederick Cancer Research and Development Center (Frederick, MD). Study animals were provided NTP-2000 open formula mean diet (Ziegler Brothers, Inc., Gardners, PA) and tap water (via automatic watering system) ad libitum. Animals were quarantined for approximately two weeks prior to the start of the studies and were approximately 8 weeks (rats and mice) or 7 weeks old (hamsters) on the first day of dosing. Study animals were distributed randomly into groups of approximate initial mean body weight and identified by tail tattoo (rats and mice) or ear tag (hamsters). Rats were housed five animals per cage. Mice and hamsters were housed individually. The animal room was maintained at a temperature of 72 ± 3°C, a relative humidity of 50 ± 15%, a light/dark cycle of 12 hours (fluorescent light) and ≥10 air changes per hour.

### 2.3. Study Design

Core study animals were fed diets containing 0, 10, 100, 1,000, 8,000, or 16,000 ppm (rats), 0, 10, 100, 1,000, 4,000, or 8,000 ppm (mice), or 0, 100, 1,000, 6,000, 12,000, or 24,000 ppm (hamsters) gemfibrozil for 14 weeks (*N* = 10). Additional groups of animals were designated as special study animals (*N* = 15) and were fed diets at the same concentrations for up to 13 weeks. For each species, the highest exposure concentration was based on the estimated maximum tolerated dose; in hamsters, the NTP conducted a 14-day study prior to selecting exposure concentrations for the 90-day study. Feed consumption by core study animals was recorded weekly. Core and special study animals were weighed initially, weekly, and at the end of the studies. Clinical findings were recorded weekly for core and special study animals. Other endpoints were determined as indicated below.

### 2.4. Clinical Chemistry

Blood for clinical chemistry was collected from special study animals on day 34 (*N* = 5) and from core study animals at the end of the studies (*N* = 10); animals were not fasted prior to blood collection. The animals were anesthetized with a mixture of carbon dioxide and oxygen, and blood was withdrawn by cardiac puncture and placed in collection tubes devoid of anticoagulant. The samples were allowed to clot and were then centrifuged; the serum was removed and stored at −70°C until analysis. The following clinical chemistry endpoints were measured in rats and hamsters: alanine aminotransferase (ALT), alkaline phosphatase (ALP), sorbitol dehydrogenase (SDH), and bile acids; mice were not evaluated for liver biomarkers due to limited serum availability. Cholesterol and triglycerides were measured in rats, mice, and hamsters.

### 2.5. Liver Histopathology and Weights

Following necropsy of both core and special study animals, the liver was weighed. Livers were then fixed and preserved in 10% neutral buffered formalin, trimmed and processed, embedded in paraffin, sectioned at 5-6 microns, and stained with hematoxylin and eosin for histopathological evaluation. Liver histopathology was conducted on all core study rats, mice (except 10 ppm), and hamsters. The histopathological findings were subjected to a rigorous pathology peer review including an NTP Pathology Working Group (PWG); the final diagnoses represent a consensus of peer review pathologist and the PWG. Details of these review procedures have been described by Maronpot and Boorman [45] and Boorman et al. [46].

### 2.6. Hepatocyte and Peroxisome Proliferation

On study days 1, 29, and 85, five special study rats, mice, and hamsters per group were implanted subcutaneously with osmotic minipumps (Model 2001, Alza Corp., Palo Alto, CA) prefilled with a 30 mg/mL solution of 5-bromo-2′-deoxyuridine (BrDU; Sigma Chemical Company, St. Louis, MO) in 0.01 N sodium hydroxide. The pumps were incubated in phosphate-buffered saline at 37°C for at least 4 hours and then implanted between 1300 and 1600 hours in animals anesthetized with 2% isoflurane via inhalation. The exact time of implantation in each animal was recorded. After 5 days (116 ± 3 hours) of BrDU exposure, the livers were evaluated for incorporation of BrDU. Approximately half of the left, right median, and anterior right lobes were fixed in 10% neutral buffered formalin for 48 hours; the remaining tissue was frozen in liquid nitrogen. The formalin-fixed liver samples, as well as a transverse section of duodenum included as an internal control, were embedded in paraffin; tissues not embedded after 48 hours of fixation were transferred to 70% ethanol. Two serial sections of each tissue were made; one slide was used for histopathologic examinations, and the second slide was stained with anti-BrDU antibody. Cell proliferation (labeled hepatocytes as a percentage of total hepatocytes) was measured by examining 2,000 hepatocyte nuclei from the left liver lobe. 

A sample of the left liver lobe was collected from the BrDU animals and reserved for peroxisome proliferation analyses; approximately 1 g (rat and hamster) or 0.5 g (mouse) portions of the liver samples were prepared and analyzed for peroxisome proliferation. Peroxisome proliferation was determined in duplicate tissue extractions by measuring *β*-oxidation, catalase activity, and nonspecific carnitine acetyltransferase activity. Peroxisomal *β*-oxidation was estimated by two methods: direct measurement of acyl coenzyme A oxidase activity [47] and measurement of the *β*-oxidation spiral [48]. Nonspecific carnitine acetyltransferase activity was estimated by the method of Gray et al. [49, 50]. Peroxisomal catalase activity was estimated by a method derived from those of Van Lente and Pepoy [51] and Yasmineh et al. [52]. Protein concentrations were measured using the bicinchoninic method with bovine serum albumin as the standard [53]; commercially available reagents were used.

### 2.7. Statistical Methods

The Fisher exact test [54], a procedure based on the overall proportion of affected animals, was used to determine the significance of lesion incidence. Organ and body weight data, which historically have approximately normal distributions, were analyzed with the parametric multiple comparison procedures of Dunnett [55] and Williams [56, 57]. Clinical chemistry and peroxisomal and hepatocyte proliferation data, which have typically skewed distributions, were analyzed using the nonparametric multiple comparison methods of Shirley [58] (as modified by Williams, [59] ) and Dunn [60]. Jonckheere's test [61] was used to assess the significance of the dose-related trends and to determine whether a trend-sensitive test (Williams' or Shirley's test) was more appropriate for pairwise comparisons than a test that does not assume a monotonic dose-related trend (Dunnett's or Dunn's test). Prior to statistical analysis, extreme values identified by the outlier test of Dixon and Massey [62] were examined by NTP personnel, and implausible values were eliminated from the analysis.

## 3. Results and Discussion

### 3.1. In Life Toxicity

All core study rats, mice, and hamsters survived to the end of the study. Final mean body weight gains of rats, mice, and hamsters were decreased by greater than 10% relative to controls at the highest two concentrations in rats and at the highest concentration in mice and hamsters (Table 1). Although initially reduced at 8,000 and 16,000 ppm (Table 1), feed consumption by exposed rats was similar to that by the controls by the end of the study (consumption was similar after week 2; data not shown). Feed consumption by mice and hamsters was generally similar to those by the controls; however, accurate estimates of food consumption were difficult to obtain due to extensive scattering of feed. Average daily doses that resulted from exposure to gemfibrozil are shown in Table 1. Doses ranged from 0.6–1300 mg/kg in rats, 1.9–2100 mg/kg in mice, and 7–2000 mg/kg in hamsters. No chemical-related clinical findings were observed in rats. Thinness was observed in mice (8,000 ppm) and in hamsters (2,000 and 24,000 ppm). The lack of decreased food consumption or signs of overt toxicity suggests that the decreased weight gains of exposed animals were due to alterations in lipid metabolism; similar findings were reported for Wy-14,643 [36].

### 3.2. Clinical Chemistry Analysis

Clinical chemistry data are presented for rats, mice, and hamsters in Table 2; mice were not evaluated for liver biomarkers due to limited serum availability.

In rats, there was a treatment-related increase (approximately 1.8-fold) in serum alanine aminotransferase activity at the highest concentration on day 34. By week 13, increases (ranging between 1.4- to 2.9-fold) in alanine aminotransferase activity occurred at the top three concentrations. Additionally, increases in sorbitol dehydrogenase activity at the highest three concentrations ranged from 1.8- to 4.7-fold. The increases in serum alanine aminotransferase and sorbitol dehydrogenase activities observed in rats would suggest a treatment-related hepatocellular effect or injury, similar to that observed for the potent peroxisome proliferators Wy-14,643 [36]. Increases in alkaline phosphatase activity and bile salt concentration, suggestive of a cholestatic event, occurred at day 34 and week 13 at the highest three concentrations. For both variables, the increases appeared to be dose-related, ranging between 1.4- to 2.3-fold for alkaline phosphatase and 2.4- to 6.7-fold for bile salts. On day 34, dose-related increases in serum cholesterol concentration occurred at the highest three concentrations; the increases were modest, ranging from 1.3- to 1.7-fold. By week 13, increases in cholesterol concentration (ranging between 1.4- to 1.9-fold) occurred in all but the lowest dose group. Conversely, at week 13, triglyceride concentration decreased by approximately 50% at the highest two concentrations.

In mice exposed for 13 weeks, a slight (20–30%) treatment-related increase in cholesterol concentration occurred at the highest three concentrations. Triglyceride concentrations, however, were decreased at the two highest concentrations; the decrease was dose-related at 35 and 44% in the 4000 and 8000 ppm dose groups, respectively. 

In hamsters, increases in bile salt concentration, suggestive of a cholestatic event, occurred on day 34 and at week 13 at the highest three concentrations; the increases appeared to be dose-related, ranging between 1.7- to 7.7-fold. Alkaline phosphatase activity, another marker of cholestasis, however, was decreased at both time points at the highest three concentrations; the decreases were modest ranging between 13 to 28%. At both time points, triglyceride concentration was increased. At day 34, treatment- but not dose-related increases in serum triglyceride concentration occurred in all groups except the lowest concentration; the increases ranged from 1.2- to 1.6-fold. By week 13, triglyceride concentration was increased (1.9-fold) only at the highest concentration. There were no changes in cholesterol concentrations. 

There was a clear and interesting difference between the species regarding the serum lipid (triglycerides and cholesterol) lowering effect of gemfibrozil. Rats and mice had decreases in triglycerides but increases in cholesterol concentration whereas hamsters had increases in serum triglycerides and no effect on cholesterol concentrations. The more potent peroxisome proliferator Wy-14,643 [36] had no effect on cholesterol or triglycerides in rats, caused decreases in triglycerides and increases in cholesterol (similar to gemfibrozil in both rats and mice) in mice, and caused decreases in serum cholesterol and triglycerides in hamsters. Hamsters are a better model for human lipoprotein metabolism that rats or mice, as hamsters, like humans, make cholesterol ester transfer protein (CETP) [16, 63]. In addition, hamsters have a similar hepatic sterol synthesis rate to humans; the rate is much higher in rats and mice [64]. It is unclear why lipid-lowering effects were not observed in hamsters following exposure to gemfibrozil in the present study.

### 3.3. Liver Histopathology and Weights

The incidence of hepatocyte cytoplasmic alteration was significantly increased in all exposed groups of rats and in mice exposed to 1000 ppm or greater (Table 3). The severity of this lesion was increased in rats exposed to 100 ppm or greater and in mice exposed to 4000 or 8000 ppm. A dose-related increase in severity was observed in both rats and mice. Hepatocyte cytoplasmic alteration was characterized by prominently increased cytoplasmic granularity and eosinophilia with some evidence of hepatocyte enlargement in severe cases. This change was generally diffuse but in some cases, the distribution was centrilobular to midlobular and of minimal severity. The granularity observed in the hepatocytes was considered consistent with the known hepatocellular appearance of peroxisome proliferation in the liver. Hepatocyte cytoplasmic alteration was not observed in hamsters, indicating a lack of morphological evidence of peroxisome proliferation; however, hepatic glycogen depletion was significantly increased in all exposed groups and increased in severity at the highest concentration (Table 3). Glycogen depletion was characterized by a decrease or absence of clear vacuoles in the cytoplasm of hepatocytes. The glycogen content of the liver is variable and may fluctuate depending on the physiological state of rodents. While glycogen depletion is commonly seen in animals that have been fasted, it may also be observed due to the pharmacologic or toxic effects of xenobiotic exposure. 

Absolute and relative liver weights were recorded in core (data not shown) and special study animals. Table 4 presents the relative liver weight data for special study animals on day 6, day 34, and week 13. In all three species, the maximum increase in relative liver weight was observed at week 13. At all time points, the relative liver weights of rats exposed to 100 ppm or greater were significantly increased. On day 6, the largest increase was observed at 8000 ppm, while the increases at 1000 ppm and 16000 ppm were similar. In mice, relative liver weights were increased at all durations at the highest two exposure concentrations and at all concentrations on day 34. In hamsters, more modest, but significant increases were observed at the highest two concentrations on day 34 and week 13. The largest increases in relative liver weight were observed in rats (up to 2.8-fold) and the smallest increases were in hamsters (up to 1.3-fold); liver weights in mice were increased at up to 1.7-fold.

### 3.4. Hepatocyte and Peroxisome Proliferation

There were significant increases in hepatocyte cell proliferation, measured as BrdU labeling of hepatocytes, in rats at all exposure durations (Table 5). Cell proliferation was increased in all exposed groups of rats on day 6. The greatest increases were observed in the 1000 ppm (9.7 fold) and 8000 ppm (7.0 fold) groups, while the increases at 100 ppm (2.5 fold) and 16000 ppm (1.9 fold) were similar. This pattern, which is similar to that observed for relative liver weight on day 6, was not observed at the day 34 and week 13 exposure durations, as the greatest increases were at the highest concentration at these durations. The magnitude of the maximum increase in cell proliferation was less with increasing exposure duration (9.7-fold on day 6, 5.4-fold on day 34, and 3.0-fold at week 13). There were no biologically significant increases in hepatocyte proliferation in mice or hamsters. The lack of an increase in mice was noteworthy, given that increases in other endpoints were observed in both rats and mice. In the NTP studies of Wy-14,643, increased hepatocyte proliferation was observed in all three species at all three exposure durations, with greater increases in rats and mice relative to hamsters [36]. In rats and hamsters, the magnitude of the response was lower at longer durations; however, the response was sustained in mice. In a feed study evaluating hepatocyte proliferation with Wy-14,643 and DEHP, at exposure durations out to one year, a sustained proliferative response was observed with Wy-14,643, but not DEHP [30]; this sustained proliferation with Wy-14,643 is likely reflective of its potency. The lack of a sustained proliferative response in rats with gemfibrozil in the present study is similar to that observed with DEHP and other peroxisome proliferators, indicating less potency relative to Wy-14,643. 

Peroxisomal enzyme activities are shown in Table 6. In rats, mice, and hamsters, Acyl CoA Oxidase, *β*-oxidation, and carnitine acetyl transferase were generally increased with increasing concentration; however, the effect was not as pronounced in hamsters. In rats and mice, the greatest increases relative to controls were generally observed at week 13. In rats, these enzymes were increased at 100 ppm or greater by week 13. In mice, these enzymes were generally increased at the highest three concentrations. In hamsters, Acyl CoA oxidase and carnitine acetyltransferase were increased at the highest two concentrations at all durations and at the highest three concentrations on day 34 and week 13 (Acyl CoA oxidase) or day 6 (carnitine acetyltransferase). *β*-oxidation was increased only at the top two concentrations on day 6. Maximum increases in Acyl CoA oxidase and carnitine acetyltransferase were similar between rats and mice, while the increase in *β*-oxidation in rats was much greater than in mice. In general, increases in catalase were observed only at higher concentrations and longer durations relative to the other enzymes in rats and mice; the lower induction of catalase relative to hydrogen peroxide generating enzymes is consistent with previous reports. Increases in catalase were not observed in hamsters. The greater observed increases in hydrogen peroxide generating enzymes relative to increases in catalase, which removes hydrogen peroxide, are consistent with previous studies on peroxisome proliferators, supporting the hypothesis that hepatocarcinogenesis may arise due to a net increase in hydrogen peroxide and subsequent oxidative stress. A generally similar pattern of increased enzyme activities was observed with Wy-14,643 [36] except that responses occurred at lower concentrations.

### 3.5. Comparison of Results with Gemfibrozil Cancer Bioassay

In a previous cancer bioassay of gemfibrozil [12], male and female albino CD rats and CD-1 mice were exposed to 0, 30, or 300 mg/kg for 104 weeks (rats) or 78 weeks (mice). The authors stated that gemfibrozil was a liver carcinogen in male rats, but not in female rats or in mice of either sex. In rats, there was a clear and significant increase in benign liver neoplastic nodules at 300 mg/kg and an increased number of liver carcinomas at both 30 and 300 mg/kg. In mice, there was a significant increase in hepatocellular carcinomas at 30 mg/kg, but not 300 mg/kg. In the present study, a dose of 300 mg/kg would result from exposure to between 1000 (60 mg/kg) and 8000 (510 mg/kg) ppm in rats and 1000 (210 mg/kg) and 4000 (920 mg/kg) ppm in mice. At these concentrations, significant increases in hepatocyte cytoplasmic alteration, relative liver weights, hepatocyte proliferation (rats only), and hydrogen peroxide producing peroxisomal enzyme activities were increased. In general, despite the fact that the dose range was higher in mice than in rats, there were greater increases in these endpoints in rats; this is especially the case with hepatocyte proliferation, which was not increased in exposed mice. Thus, it appears that the species susceptibility to liver tumors correlates with that of peroxisome-proliferation-related hepatic effects. However, the observed differences in response may be the result of different exposure durations.

### 3.6. Studies by Investigators Utilizing NTP Tissues

Several investigators utilized tissues from the NTP peroxisome project studies to evaluate mechanisms of peroxisome proliferator-induced hepatocarcinogenicity with selected compounds. These studies typically evaluated oxidative stress-related mechanisms of action. Differences in species susceptibility between rats and hamsters were observed with several endpoints, including selenium dependent glutathione peroxidase activity, which was increased in hamsters and decreased in rats following exposure to Wy-14,643, GEM, and DBP [38]; activation of NFkappaB, occurred in rats primarily with Wy-14,643 but to a lesser extent with Gem and DBP, but not in hamsters [39]; and polymerase-*β*, Ref-1, and PNCA were increased in rats but either observed at trace levels (polymerase-*β*) or decreased (Ref-1 and PCNA) in hamsters, following exposure to Wy-14,643 [42]. In contrast, some endpoints did not reflect species differences, including glutathione-S-transferase and glutathione reductase activities following exposure to Wy-14,643 and DBP [38], activation of several redox-sensitive transcription factors, including AP-1 early growth response gene 1 and heat shock factors 1 and 2 following exposure to Wy-14,643, GEM or DBP [40], expression of the proapoptotic protein Bax following exposure to Wy-14,643, GEM, and DBP [42], and antioxidant capacities with dibutyl phthalate, gemfibrozil, or Wy-14,643 [41]. Exposure of rats and mice to Wy-14,643 increased the expression of several base excision repair enzymes, but not the expression of enzymes that are not involved in the repair of oxidative DNA damage [44]. The other compounds induced weaker or no increases in the expression of these enzymes. In another study, WY, Gem, DBP, and 2,4-D were evaluated for their ability to alter the methylation and expression of the c-myc protooncogene in mice [43]. All four peroxisome proliferators caused hypomethylation of the c-myc gene in the liver, while only Wy-14,643 increased the level of c-myc protein. Collectively, these studies provide some insight regarding oxidative stress-related mechanisms of peroxisome proliferators and species differences in susceptibility.

### 3.7. Comparison of PPAR*α*-Mediated Effects in Rodents and Humans

Recent studies by Gonzalez and colleagues using humanized PPAR*α* mice have provided some information on the mechanism of PPAR*α*-hepatocarcinogenicity in rodents and species differences between rodents and humans following exposure to rodent peroxisome proliferators [25, 26, 32, 34, 35]. Hepatocellular proliferation and neoplasms were observed in wild type, but not humanized PPAR*α* mice. A proposed mechanism of the hepatic proliferative effects involves downregulation of let-7C miRNA, resulting in the increased expression of c-myc, which in turn results in increased hepatocellular proliferation and tumors [26, 34, 35]. In contrast, these biochemical and morphologic effects are not observed in humanized PPAR*α* mice. In mice with both receptor types, induction of genes involved in peroxisomal and mitochondrial *β*-oxidation were observed. These data may explain the difference in PPAR*α*-mediated effects between rodents and humans.

Several studies evaluated the pleiotropic responses to prolonged (from 14 days up to 13 weeks) oral administration of relatively high doses of peroxisome proliferators (500 to 2500 mg/kg) in several species of nonhuman primates [65–67]. In contrast to results found in rodents, no significant increases in liver weight, induction of peroxisomal enzymes, or proliferation of peroxisomes were reported. Studies that examined patients treated with relatively more potent PPs (e.g., clofibrate, gemfibrozil, or fenofibrate), for prolonged periods of time (i.e., years) are more consistent with the idea that humans do not exhibit peroxisome proliferation in response to exposure to PPs. Similar to findings made in hemodialysis patients, a marginal 50% increase in liver peroxisome number, but not in peroxisome volume, is reported in humans treated with clofibrate [68]. In contrast, the majority of studies examining the effect of PP administration in humans have consistently shown no change in hepatic peroxisome proliferation in liver (reviewed in [24]).

There are no known reports of long-term carcinogenesis studies with PPs in nonhuman primates. Several large epidemiological studies that examined the relationship between chronic treatment with lipid-lowering PPs gemfibrozil and clofibrate did not find an association with liver cancer (reviewed in [24]). Collectively, human epidemiological studies have not shown an association between liver cancer and treatment with PPs [69].

The molecular mechanism by which hypolipidemic fibrates and antidiabetic thiazolidinediones exert their therapeutic effect in humans is similar to the way peroxisome proliferators exert their toxicity in rodents, namely, by activation of the PPAR family of receptors. In response to exposure to a PP chemical, the mRNA and protein levels of numerous enzymes are increased in rodents, including the enzymes in the peroxisome per se but also microsomal cytochrome CYP4A. Primary organs involved in this pleiotropic response are liver, kidney, and heart. A receptor responsible for activating these diverse effects was identified, termed the peroxisome proliferator-activated receptor (PPAR) and was demonstrated to belong to the nuclear receptor superfamily that includes the estrogen, progesterone, and retinoic acid receptors. Members of the PPAR family of receptors include PPAR*α*, PPAR*β*/*δ*, and PPAR*γ*, which have different tissue distributions, abundances and functions in lipid metabolism during different stages of development. PPAR*γ* mRNA has been detected in greatest amounts in human heart, placenta, lung, and kidney, but has also been identified in human prostate, testis, and ovary [70, 71]. 

PPAR*α* mediates gene activation through binding to a DNA response element (PPRE) (a DR-1 response element) upstream from all genes that are known to respond to PPs. These include genes in the peroxisome mentioned above as well as cytochromes CYP4A and fatty acid binding protein. The other members of the PPAR superfamily (PPAR*β*/*δ* and *γ*) bind to and activate similar PPRE but in different tissues. PPAR-ligand complex binds to the PPRE upstream of the LPL and Apo A I and -II genes in humans, whereas it binds upstream and activates different genes in rodents, namely, those genes responsible for the peroxisome proliferation response. The increased lipoprotein lipase and apolipoprotein (apo) A-I and apoA-II induction increase plasma HDL and increase triglyceride mobilization. In rats PPAR*α* activation decreases apoA-I and apoA-II gene expression and lowers plasma HDL [72]. In humans, HDL cholesterol is elevated after fibrate treatment due to increased lipolysis of triglyceride-rich lipoproteins and redistribution of lipid components to HDL. 

Although the PPRE is almost identical in rodents (TGCCCTTCCCCC) and humans (TGCCCTTCCCCC), the location in the genome of the PPRE is different across species resulting in vastly different genes expressed following activation of the PPAR family. 

The human receptor appears to be activated by certain fatty acids and eicosanoids and thiazolidinedione antidiabetic drugs, although it appears to be only weakly activated by classical PPs such as, Wyeth-14,643 nafenopin and clofibric acid [71]. Endogenous ligands for PPARs include most straight-chain fatty acids, substituted fatty acids, and the acyl-CoA esters of fatty acids, and arachidonic acid derived prostaglandins and eicosanoids [73]. 

In humans, like rodents, fibrate drugs used in the treatment of hyperlipidemia are thought to activate PPAR*α* in the liver. However, unlike rodents, activation of PPARa in humans does not result in peroxisome proliferation but results in increased apolipoprotein A-II and lipoprotein lipase transcription, and reduced apolipoprotein C-III, which is key to their mechanism of action to lower serum triglycerides [74–76] as well as induction of fatty acid transport protein and acyl-CoA synthetase [77]. (Apo C-III is a major component of very low-density lipoproteins (VLDL) and inhibits lipoprotein lipase and inhibits clearance of lipoproteins by the liver). 

The antidiabetic agents in the thiazolidinediones activate human PPAR*γ* in adipose tissue where lipoprotein lipase expression is also increased. LPL is transcriptionally activated and results in increased lipolytic activity and a decrease in serum triglycerides in humans without an increase in peroxisome activity seen in rodents, again due to the location of the PPAR*γ* response element upstream of the LPL gene [75].

## 4. Conclusions

The present NTP studies confirm the induction of hepatomegaly and hepatocyte and peroxisome proliferation and alteration of lipids following exposure to gemfibrozil. Similar to NTP studies with Wy-14,643 [36], these studies also present data on hamsters, which were considered, like humans, to be nonresponsive to PPAR*α*-mediated effects on hepatic and peroxisome proliferation, similar to primates and humans. Based on these data, it is apparent that rats are most responsive to the hepatic effects of gemfibrozil, while mice are intermediate and hamsters are the least responsive; however, the increases in peroxisomal enzymes indicate peroxisome proliferation is induced in hamsters. In all three species, the pattern of peroxisomal enzyme is consistent with previous reports, with greater increases in hydrogen peroxide-generating enzymes compared to catalase. The greater sensitivity to the induction of hepatic peroxisome and hepatocellular proliferation in rats compared to mice may explain the differences in liver carcinogenicity between the two species observed in a previous study. Gemfibrozil produced alterations of lipid metabolism in each species; the effects and rats and mice were similar and distinct from hamsters.

It is clear from several investigators that humans possess a functional PPAR family of receptors. It is also clear that they regulate different genes relative to the receptor family in rodents, and that the human PPAR receptor is activated by xenobiotic drugs and chemicals. What is less clear is the relative potency of phthalates to activate the hPPAR family compared to therapeutic agents as well as compared to endogenous activators, and what such activation, if any, would result that may have deleterious effects in humans [11]. Indeed, in two recent reviews of the medical significance of PPARs, it was reported that since PPAR does not induce peroxisomes in humans the term peroxisome proliferator per se in a medical context is a misnomer ([21, 78] and references contained therein). An excellent review of the mechanism of action of fibrates in humans was published recently [79]. Recently published studies using humanized PPAR*α* mice have provided mechanistic insights into the observed hepatocarcinogenicity in rodents and on differences between rodents and humans [25, 26, 32, 34, 35].

## Figures and Tables

**Table 1:** Survival, body weights, average daily doses, and feed consumption in male core study Harlan Sprague Dawley rats, B6C3F1 mice, and Syrian hamsters following exposure to gemfibrozil in feed for 14 weeks^a^.

Dose (ppm)	Survival^a^	Initial Body Weight^b^ (g)	Final Body Weight^b^ (g)	Body Weight Change^b^ (g)	Final Body Weight (% Con)	Wk 1 Feed Consumption (g/animal/day)	Week 13 Feed Consumption (g/animal/day)	Average Daily Dose (mg/kg)
Rats								
0	10/10	236 ± 3	436 ± 5	201 ± 3	—	20.9	19.1	—
10	10/10	236 ± 2	424 ± 9	189 ± 8	97	19.9	18.6	0.6
100	10/10	235 ± 2	432 ± 8	197 ± 7	99	21.5	20.8	6
1000	10/10	237 ± 3	410 ± 16	173 ± 14*	94	20.2	20.1	60
8000	10/10	231 ± 3	350 ± 9**	119 ± 7**	80	13.0	19.3	510
16000	10/10	237 ± 2	286 ± 7**	49 ± 6**	66	7.5	22.8	1300

Mice								
0	10/10	22.1 ± 0.1	33.7 ± 0.6	11.6 ± 0.5	—	5.3	6.4	—
10	10/10	22.1 ± 0.2	35.5 ± 0.8	13.4 ± 0.6	105	6.0	5.4	1.9
100	10/10	22.1 ± 0.3	35.1 ± 0.7	13.0 ± 0.7	104	5.8	5.5	19
1000	10/10	21.2 ± 0.2	34.4 ± 0.6	12.2 ± 0.5	102	6.0	6.2	210
4000	10/10	21.5 ± 0.2	32.0 ± 0.3	10.5 ± 0.3	95	5.8	7.4	920
8000	10/10	22.0 ± 0.2	28.8 ± 0.2**	6.7 ± 0.3**	85	5.9	7.6	2100

Hamsters								
0	10/10	78 ± 1	115 ± 2	37 ± 2	—	8.8	7.3	—
100	10/10	79 ± 2	125 ± 4	46 ± 4	108	8.0	7.4	7
1000	10/10	77 ± 2	118 ± 3	40 ± 2	102	7.8	7.0	80
6000	10/10	76 ± 2	120 ± 4	44 ± 4	104	7.9	6.8	480
12000	10/10	78 ± 2	109 ± 5	30 ± 5	95	7.6	7.2	970
24000	10/10	79 ± 2	99 ± 4**	20 ± 3**	86	9.7	6.3	2000

*Significantly different (*P* ≤ .05) from the control group by Williams' test; **P ≤ .01; ^a^Number of animals surviving at 3 months/number initially in group; ^b^Mean ± standard error.

**Table 2:** Clinical chemistry data for male Harlan Sprague Dawley rats, B6C3F1 mice, and Syrian hamsters following exposure to gemfibrozil in feed for 34 days (special study) or 14 weeks (core study)^a^.

Rats	0 ppm	10 ppm	100 ppm	1000 ppm	8000 ppm	16000 ppm
*n*						
Day 34	5	5	5	5	5	5
Week 14	10	10	10	10	10	10
ALT (IU/L)						
Day 34	70 ± 5	65 ± 2	66 ± 5	67 ± 2	73 ± 4	126 ± 13*
Week 14	75 ± 6	71 ± 3	74 ± 5	101 ± 8**	219 ± 42**	178 ± 16**
SDH (IU/L)						
Day 34	21 ± 3	16 ± 2	24 ± 4	27 ± 4	26 ± 1	28 ± 7
Week 14	30 ± 4	31 ± 3	38 ± 3	54 ± 9**	141 ± 30**	86 ± 18**
ALP (IU/L)						
Day 34	706 ± 54	642 ± 13	802 ± 32	999 ± 66*	1,227 ± 66**	1,541 ± 211**
Week 14	486 ± 19	452 ± 13	560 ± 47	788 ± 53**	697 ± 30**	1112 ± 50**
Bile Salts (*μ*mol/L)						
Day 34	29.4 ± 5.4	26.4 ± 7.2	41.4 ± 6.5	71.6 ± 8.1*	136.2 ± 11.7**	192.8 ± 27.7**
Week 14	55.3 ± 8.2	38.4 ± 2.9	54.9 ± 9.1	79.9 ± 10.4	133.4 ± 19.7**	223.4 ± 18.0**
Cholesterol (mg/dL)						
Day 34	131 ± 8	122 ± 5	150 ± 5	172 ± 5**	197 ± 11**	223 ± 14**
Week 14	120 ± 4	122 ± 4	195 ± 9**	172 ± 8**	212 ± 5**	231 ± 5**
Triglycerides (mg/dL)						
Day 34	99 ± 10	91 ± 7	91 ± 8	77 ± 7	73 ± 12	84 ± 9
Week 14	124 ± 13	121 ± 7	104 ± 13	105 ± 7	72 ± 6**	58 ± 3**

Mice	0 ppm	10 ppm	100 ppm	1000 ppm	4000 ppm	8000 ppm

Cholesterol (mg/dL)						
Day 34	159 ± 8	144 ± 5	146 ± 2	144 ± 2	180 ± 6	194 ± 3
Week 14	175 ± 3	190 ± 5	184 ± 4	212 ± 6**	219 ± 5**	216 ± 6**
Triglycerides (mg/dL)						
Day 34	142.6 ± 13.2	156.0 ± 1.47	154.8 ± 14.8	160.4 ± 5.6	122.8 ± 13.6	117.6 ± 6.6
Week 14	181.6 ± 19.8	182.1 ± 15.6	158.8 ± 10.6	134.1 ± 8.7	116.8 ± 8.8**	100.9 ± 6.0**

Hamsters	0 ppm	100 ppm	1000 ppm	6000 ppm	12000 ppm	24000 ppm

ALT (IU/L)						
Day 34	67 ± 13^b^	76 ± 14^b^	48 ± 1^b^	76 ± 7^b^	62 ± 11	58 ± 14
Week 14	73 ± 3	73 ± 10	86 ± 13	63 ± 6	71 ± 5	81 ± 10
SDH (IU/L)						
Day 34	57 ± 11^b^	59 ± 8	39 ± 2^b^	62 ± 5^b^	51 ± 9	44 ± 4
Week 14	51 ± 2	59 ± 6	73 ± 21	52 ± 4	53 ± 3	65 ± 11
ALP (IU/L)						
Day 34	265 ± 17^b^	277 ± 21^b^	241 ± 14^b^	215 ± 14^b∗^	216 ± 12*	190 ± 13**
Week 14	202 ± 7	192 ± 9	177 ± 15	177 ± 7	149 ± 10**	152 ± 15**
Bile Salt (*μ*mol/L)						
Day 34	11.3 ± 0.5^b^	10.0 ± 1.1	9.5 ± 0.9^b^	11.8 ± 1.4^b^	19.0 ± 1.4	42.4 ± 8.7**
Week 14	8.9 ± 0.7	11.3 ± 1.3^c^	11.1 ± 1.8^d^	19.5 ± 7.3	26.8 ± 5.7*	68.6 ± 10.6**
Cholesterol (mg/dL)						
Day 34	149 ± 6	143 ± 8	133 ± 5	156 ± 9	148 ± 5	152 ± 7
Week 14	149 ± 3	151 ± 8	139 ± 5	129 ± 8	153 ± 6	153 ± 7
Triglycerides (mg/dL)						
Day 34	163 ± 13	171 ± 11	240 ± 27**	263 ± 16**	192 ± 8**	258 ± 19**
Week 14	201 ± 10	210 ± 20	203 ± 17	217 ± 21	214 ± 26	375 ± 42**

*Significantly different (*P* ≤ .05) from the control group by Dunn's or Shirley's test; **P ≤ .01; ^a^Mean ± standard error, statistical tests were performed on unrounded data; ^b^ *n* = 4; ^c^ *n* = 8; ^d^ *n* = 9.

**Table 3:** Incidence and severity of liver histopathologic lesions in male core study Harlan Sprague Dawley rats, B6C3F1 mice, and Syrian hamsters following exposure to gemfibrozil in feed for 14 weeks^a^.

Rats	0 ppm	10 ppm	100 ppm	1000 ppm	8000 ppm	16000 ppm
Liver, Cytoplasmic Alteration	0	7^b∗∗^(1.0)^c^	10** (2.0)	10** (3.0)	10** (4.0)	10** (4.0)

Mice	0 ppm	10 ppm	100 ppm	1000 ppm	4000 ppm	8000 ppm

Liver, Cytoplasmic Alteration	0	NE	0	7** (1.0)	10** (2.6)	10** (3.0)

Hamsters	0 ppm	100 ppm	1000 ppm	6000 ppm	12000 ppm	24000 ppm

Liver, Glycogen Depletion	0	5^d∗∗^ (1.0)	4* (1.0)	8** (1.0)	9** (1.0)	10** (2.8)

*Significantly different (*P* ≤ .05) from the control group by the Fisher exact test; **P ≤ .01; NE = not examined; ^a^ *n* = 10; ^b^Incidence; ^c^Mean  severity: 1 = minimal, 2 = mild, 3 = moderate, 4 = marked; ^d^ *n* = 9.

**Table 4:** Relative liver weights in male special study Harlan Sprague Dawley rats, B6C3F1 mice, and Syrian hamsters following exposure to gemfibrozil in feed for 6 days, 34 days, or 13 weeks^a,b^.

Rats	0 ppm	10 ppm	100 ppm	1000 ppm	8000 ppm	16000 ppm
Day 6	40.720 ± 1.154	43.574 ± 0.995	49.162 ± 1.292**	55.277 ± 1.702**	59.642 ± 1.612**	54.037 ± 0.718**
Day 34	36.76 ± 0.540	37.50 ± 0.583	45.444 ± 0.693**	53.942 ± 1.449**	68.603 ± 2.391**	75.290 ± 1.296**
Week 13	31.627 ± 1.168	33.179 ± 0.357	39.391 ± 0.866**	48.137 ± 0.523**	74.370 ± 1.212**	86.782 ± 1.678**

Mice	0 ppm	10 ppm	100 ppm	1000 ppm	4000 ppm	8000 ppm

Day 6	51.954 ± 0.788	50.291 ± 1.108	51.703 ± 2.524	54.417 ± 1.465	64.524 ± 1.391**	76.920 ± 0.441**
Day 34	41.768 ± 1.153	45.376 ± 0.687**	47.546 ± 0.742**	51.955 ± 0.327**	63.724 ± 1.036**	72.806 ± 0.749**
Week 13	41.152 ± 0.390	42.606 ± 0.862	41.689 ± 0.737	43.299 ± 1.153	60.370 ± 01.303**	71.208 ± 1.235**

Hamsters	0 ppm	100 ppm	1000 ppm	6000 ppm	12000 ppm	24000 ppm

Day 6	5.447 ± 0.286	5.119 ± 0.195	5.463 ± 0.384	5.448 ± 0.219	5.364 ± 0.157	5.214 ± 0.272
Day 34	4.238 ± 0.196	4.292 ± 0.093	4.295 ± 0.076	4.629 ± 0.195	4.723 ± 0.126*	5.341 ± 0.106**
Week 13	3.867 ± 0.086	3.718 ± 0.121	3.864 ± 0.122	3.940 ± 0.082	4.586 ± 0.067**	5.072 ± 0.157**

*Significantly different (*P* ≤ .05) from the control group by Dunnett's or Williams' test; **P ≤ .01; ^a^Data are given as mg organ weight/g body weight (mean ± standard error); ^b^ *n* = 5.

**Table 5:** Hepatocyte proliferation (% BrdU labeled hepatocytes) in male special study Harlan Sprague Dawley rats, B6C3F1 mice, and Syrian hamsters following exposure to gemfibrozil in feed for 6 days, 34 days, or 13 weeks^a,b^.

Rats	0 ppm	10 ppm	100 ppm	1000 ppm	8000 ppm	16000 ppm
Day 6	3.740 ± 0.154	5.303 ± 0.558**	9.333 ± 1.293**	36.244 ± 1.692**	26.061 ± 3.332**	7.152 ± 0.989^c∗∗^
Day 34	0.783 ± 0.065	0.754 ± 0.143	0.679 ± 0.195	0.850 ± 0.199	1.300 ± 0.197	4.199 ± 0.867**
Week 13	0.451 ± 0.113	0.421 ± 0.061	0.461 ± 0.059	0.499 ± 0.076	0.608 ± 0.186	1.348 ± 0.142**

Mice	0 ppm	10 ppm	100 ppm	1000 ppm	4000 ppm	8000 ppm

Day 6	2.366 ± 0.345	1.414 ± 0.546	1.141 ± 0.239	1.766 ± 0.768	2.340 ± 0.842	4.449 ± 0.555
Day 34	0.896 ± 0.240	1.488 ± 0.240	1.599 ± 0.201	1.768 ± 0.385	2.097 ± 0.350	1.627 ± 0.388
Week 13	0.705 ± 0.069	1.167 ± 0.274	1.052 ± 0.157	1.240 ± 0.315	1.062 ± 0.122	0.958 ± 0.130

Hamsters	0 ppm	100 ppm	1000 ppm	6000 ppm	12000 ppm	24000 ppm

Day 6	2.042 ± 0.863	1.123 ± 0.117	4.323 ± 1.652	6.108 ± 3.669	2.572 ± 0.990	3.594 ± 1.598
Day 34	2.713 ± 0.582	2.148 ± 0.665	1.120 ± 0.249	3.932 ± 0.267	3.467 ± 0.962	1.566 ± 0.473
Week 13	1.176 ± 0.267	3.962 ± 0.848*	4.787 ± 1.085*	1.969 ± 0.169	3.625 ± 1.637	2.450 ± 0.478

**Significantly different (*P* ≤ .01) from the control group by Shirley's test; ^a^Mean ± standard  error; ^b^ *n* = 5; ^c^ *n* = 4; BrdU: bromodeoxyuridine.

**Table 6:** Hepatic peroxisomal enzyme activities in male special study Harlan Sprague Dawley rats, B6C3F1 mice, and Syrian hamsters following exposure to gemfibrozil in feed for 6 days, 34 days, or 13 weeks^a,b^.

Rats	0 ppm	10 ppm	100 ppm	1000 ppm	8000 ppm	16000 ppm
Acyl CoA oxidase (nmol DCF/minute per mg)						

Day 6	1.5 ± 0.2	1.8 ± 0.7	1.3 ± 0.2	2.0 ± 0.1	6.9 ± 0.7**	11.1 ± 1.0**
Day 34	1.8 ± 0.2	1.9 ± 0.1	2.5 ± 0.5	5.2 ± 0.4**	16.5 ± 2.6**	22.1 ± 2.4**
Week 13	1.6 ± 0.1	1.6 ± 0.1	2.9 ± 0.4*	6.8 ± 0.5**	27.2 ± 0.9**	33.2 ± 1.2**

*β*-Oxidation (Lazarow method) (nmol NADH/minute per mg)						

Day 6	1.0 ± 0.1	1.0 ± 0.1	1.0 ± 0.2	2.3 ± 0.3	12.2 ± 1.0**	19.8 ± 2.0**
Day 34	1.2 ± 0.2	1.0 ± 0.1	2.3 ± 0.3*	10.0 ± 1.3**	37.9 ± 6.0**	55.8 ± 7.0**
Week 13	1.4 ± 0.2	1.4 ± 0.1	2.4 ± 0.3*	16.9 ± 1.3**	75.2 ± 4.8**	89.9 ± 4.2**

Carnitine acetyltransferase (nmol reduced CoA/minute per mg)						

Day 6	0.8 ± 0.1	0.9 ± 0.1	1.5 ± 0.5**	2.0 ± 0.1**	11.3 ± 1.2**	15.9 ± 1.7**
Day 34	0.7 ± 0.1	0.7 ± 0.1^c^	2.3 ± 0.2**	7.0 ± 1.3**	18.6 ± 4.0**	21.8 ± 3.1**
Week 13	0.6 ± 0.1	0.8 ± 0.1	3.3 ± 0.9**	13.9 ± 2.3**	55.6 ± 5.2**	42.8 ± 5.1**

Catalase (nmol NADPH/minute per mg)						

Day 6	253 ± 17	234 ± 17	177 ± 13	162 ± 9	302 ± 11	292 ± 18
Day 34	289 ± 23	253 ± 16	222 ± 18	329 ± 29	438 ± 30*	476 ± 26*
Week 13	274 ± 28	281 ± 6.0	253 ± 22	378 ± 28*	523 ± 24**	508 ± 29**

Mice	0 ppm	10 ppm	100 ppm	1000 ppm	4000 ppm	8000 ppm

Acyl CoA oxidase (nmol DCF/minute per mg)						

Day 6	1.2 ± 0.1	1.0 ± 0.0	1.1 ± 0.1	2.5 ± 0.1*	8.2 ± 1.8**	16.5 ± 2.2**
Day 34	1.2 ± 0.1	1.4 ± 0.2	1.0 ± 0.3	2.7 ± 0.5*	8.5 ± 0.7**	15.5 ± 0.4**
Week 13	1.2 ± 0.1	1.4 ± 0.1	1.1 ± 0.1	1.7 ± 0.1*	10.9 ± 1.3**	18.0 ± 1.7**

*β*-Oxidation (Lazarow method) (nmol NADH/minute per mg)						

Day 6	0.5 ± 0.2^c^	1.0 ± 0.2	0.8 ± 0.2	1.9 ± 0.1^c∗∗^	15.3 ± 0.5^c∗∗^	30.6 ± 1.3**
Day 34	1.0 ± 0.3	1.0 ± 0.2	0.9 ± 0.1^c^	1.9 ± 0.1*	19.8 ± 1.1**	45.4 ± 1.3**
Week 13	1.2 ± 0.1^c^	1.2 ± 0.1	1.2 ± 0.2	1.9 ± 0.2	21.3 ± 0.6**	42.2 ± 1.5**

Carnitine acetyltransferase (nmol reduced CoA/minute per mg)						

Day 6	1.2 ± 0.1	1.4 ± 0.2	1.4 ± 0.1	2.6 ± 0.3**	11.1 ± 2.0**	17.2 ± 0.5**
Day 34	1.7 ± 0.2	1.3 ± 0.1	1.2 ± 0.1	2.7 ± 0.3	15.7 ± 1.3*	24.6 ± 1.4**
Week 13	1.5 ± 0.2	1.7 ± 0.1	1.8 ± 0.3	2.8 ± 0.3**	18.1 ± 0.9**	24.8 ± 1.0**

Catalase (nmol NADPH/minute per mg)						

Day 6	98.3 ± 6.2	96.8 ± 4.2	95.8 ± 2.8	96.0 ± 2.6	187.8 ± 27.4	314.7 ± 8.7**
Day 34	98.3 ± 4.0	88.1 ± 3.9	86.6 ± 3.8	89.3 ± 6.3	225.9 ± 8.8	289.2 ± 4.3**
Week 13	74.8 ± 4.4	75.4 ± 7.1	84.2 ± 10.5	78.7 ± 7.4	238.8 ± 8.4**	302.1 ± 11.3**

Hamsters	0 ppm	100 ppm	1000 ppm	6000 ppm	12000 ppm	24000 ppm

Acyl CoA oxidase (nmol DCF/minute per mg)						

Day 6	2.4 ± 0.2	2.1 ± 0.1	2.5 ± 0.2	3.1 ± 0.4	4.0 ± 0.2*	4.4 ± 0.6*
Day 34	2.1 ± 0.2	2.3 ± 0.2	2.3 ± 0.2	3.1 ± 0.2*	3.2 ± 0.3*	4.6 ± 0.4**
Week 13	2.2 ± 0.1	2.2 ± 0.1	2.1 ± 0.1	2.9 ± 0.1**	3.2 ± 0.2^c∗∗^	3.7 ± 0.4**

*β*-Oxidation (Lazarow method) (nmol NADH/minute per mg)						

Day 6	1.9 ± 0.1	1.9 ± 0.5	1.7 ± 0.1	2.2 ± 0.4^d^	2.7 ± 0.2^c^	3.0 ± 0.2**
Day 34	2.2 ± 0.1	1.8 ± 0.3	2.0 ± 0.3	2.0 ± 0.2	1.6 ± 0.3^c^	2.0^e^
Week 13	2.1 ± 0.1	2.0 ± 0.1	1.6 ± 0.3	1.6 ± 0.2	2.0 ± 0.2^c^	2.2 ± 0.5^f^

Carnitine acetyltransferase (nmol reduced CoA/minute per mg)						

Day 6	8.0 ± 0.5	8.1 ± 0.2	7.6 ± 0.6	10.8 ± 0.4**	12.8 ± 0.9**	14.1 ± 1.6**
Day 34	7.0 ± 0.2	6.1 ± 0.2	8.2 ± 0.5	7.4 ± 0.4	9.3 ± 0.4*	17.2 ± 2.1**
Week 13	6.8 ± 0.4	6.0 ± 0.2	7.5 ± 0.8	8.2 ± 0.3	10.8 ± 2.0*	14.1 ± 2.2**

Catalase (nmol NADPH/minute per mg)						

Day 6	273 ± 21	304 ± 9	249 ± 25	260 ± 37^c^	251 ± 17	226 ± 11
Day 34	302 ± 24	312 ± 22	285 ± 12	284 ± 22	258 ± 16	261 ± 34
Week 13	291 ± 19	272 ± 17	269 ± 26	270 ± 10	246 ± 14	252 ± 13^c^

**Significantly different (*P* ≤ .01) from the control group by Shirley's test; ^a^Mean ± standard  error; ^b^ *n* = 5; ^c^ *n* = 4; ^d^ *n* = 3; ^e^ *n* = 1, no standard error presented because only one sample available; ^f^ *n* = 2.
